# Hyperuricemia is Related to the Risk of Cardiovascular Diseases in Ethnic Chinese Elderly Women

**DOI:** 10.5334/gh.1102

**Published:** 2022-02-22

**Authors:** Leilei Liu, Xiao Zhang, Lian Peng, Nana Ma, Tingting Yang, Chan Nie, Linyuan Zhang, Zixuan Xu, Jun Yang, Xuejie Tang, Liubo Zheng, Tao Zhang, Feng Hong

**Affiliations:** 1 School of Public Health, the Key Laboratory of Environmental Pollution Monitoring and Disease Control, Ministry of Education, Guizhou Medical University, Guiyang 550025, China; 2 Center for Diseases Control and Prevention of Yunyan District, Guiyang 550004, China; 3 Guiyang Center for Diseases Control and Prevention, Guiyang 550003, China; 4 The Higher Education Mega Center Hospital, Guizhou Medical University, Guiyang 550025, China; 5 Guizhou Provincial People’s Hospital, Guiyang 550002, China; 6 Guiyang Public Health Clinical Center, Guiyang 550004, China

**Keywords:** Hyperuricemia, Cardiovascular Diseases, Ethnic Difference, Women, Elderly

## Abstract

**Background:**

The association between hyperuricemia (HUA) and cardiovascular diseases (CVDs) is not fully elucidated.

**Objective:**

To assess the relationship according to factors of sex and age in the Chinese ethnic groups.

**Methods:**

We performed a population–based cross-sectional study in a multi-ethnic population from southwestern China. HUA patients were identified by serum uric acid ≥7 mg/dL in men and 6 mg/dL in women. The outcome was composite prevalent CVDs, including coronary heart disease (CHD), stroke, and arrhythmia. Multivariate logistic regression analysis, estimating odds ratio (ORs) and 95% confidence intervals (CIs), were applied to evaluate the HUA–CVDs relationship.

**Results:**

We included 16,618 people (37.48% Dong, 30.00% Miao, and 32.52% Bouyei) aged 30–79 years without a reduced estimated glomerular filtration rate <60 mL/min/1.73 m^2^. CVDs developed in 250 Dong, 196 Miao, and 205 Bouyei adults. Among women, HUA was positively associated with the risk of stroke in Dong ethnicity and CVDs in Bouyei ethnicity (ORs (95% CIs) 2.02 (1.07–3.81) and 1.66 (1.06–2.59)) compared with non-HUA. In the age-specific analysis, HUA was related to the risk of CVDs (OR 2.32, 95% CI 1.00–5.38) and CHD (5.37, 1.61–17.89) among Miao people aged < median age, CVDs (1.52, 1.11–2.08) and stroke (1.67, 1.02–2.72) among Dong adults aged ≥ median age, and CVDs (1.67, 1.16–2.40) and CHD (1.77, 1.13–2.77) among Bouyei ethnicity aged ≥ median age. After stratification by sex and the median age, for women aged > 50 years, a 55% (1.55, 1.00–2.39) and 65% (1.65, 1.02–2.66) increased risk for CVDs was observed in Dong and Bouyei ethnicities.

**Conclusions:**

HUA may be related to an increased risk of CVDs among women in the Dong and Bouyei ethnic groups in China, especially women aged > 50 years.

## 1. Introduction

Cardiovascular diseases (CVDs) are the leading cause of death in the world [1], and more than half of CVDs deaths occur in Asia [2], especially China [3,4]. Global deaths from CVDs may increase approximately 1.4-fold from 2012 to 2030 [5]. A less than 25% reduced burden of CVDs was found in southwestern China over the past 30 years [6]. Therefore, the prevention of CVDs has become a major public health concern.

The association of hyperuricemia (HUA) with the risk of CVDs has been paid considerable attention in recent years [7,8,9,10,11]. However, studies to date have focused primarily on total populations, and the results were inconsistent [8,11,12,13,14,15]. Also, the sex-specific association between HUA and CVDs risk has rarely been examined and the conclusion remained controversial [7,9,10,16,17,18]. Additionally, the age-specific analysis in this area is limited [9]. Moreover, to our knowledge, other general population–based data stratified by sex and age on the relationship between HUA and the risk of CVDs are still lacking, except for a cross-sectional study that included 8,285 adults limited to a northeastern Chinese general population [9]. Furthermore, information on whether the association varies by ethnicity is not available.

To fill this evidence gap, we aimed to explore the potential effect of sex and age on the relationship between HUA and the risk of CVDs and its subtypes in the ethnic groups in Guizhou province, southwestern China, based on a cross-sectional design using data from the China Multi-Ethnic Cohort Study [19,20,21,22,23], an ongoing cohort study.

## 2. Methods

### 2.1. Study Design and Participants

Between July of 2018 and August of 2019, 18,790 participants aged 30–79 years were recruited by questionnaire interview, anthropometric and laboratory measurements from Guizhou Province of southwestern China using a multistage, stratified cluster sampling procedure based on the China Multi-Ethnic Cohort Study [19,20,21,22,23]. The three ethnic groups, including Dong, Miao, and Bouyei, were enrolled in this study.

We excluded data for individuals missing data of serum uric acid (SUA) or serum creatinine (Scr) levels (n = 1,726) or with a fasting time of less than eight hours (n = 95). We excluded participants with a reduced estimated glomerular filtration rate (eGFR) [24] <60 mL/min/1.73 m^2^ (n = 351). As a result, there was a 16,618 final analytic sample of participants (6,228 Dong, 4,986 Miao, and 5,404 Bouyei ethnic groups).

Ethics approval was obtained from the Sichuan University Medical Ethical Review Board (K2016038) and the Research Ethics Committee of The Affiliated Hospital of Guizhou Medical University (2018 [094]). All participants gave written informed consent before taking part in this study.

We defined this study as follows according to the PICOS principle: P (population): general population, I (intervention/exposure): HUA, C (comparison): non-HUA, O (outcome): CVDs, S (study design): cross-sectional study.

### 2.2. Data collection

Self-reported data on general sociodemographic information (sex, age, ethnicity, and residence), health-related behaviours (tobacco smoking, alcohol drinking weekly, total physical activity, and dietary intake frequency), and personal and family medical history of CVDs were collected by an interviewer-administered laptop-based questionnaire for each participant. Smokers were defined as people ever smoking at least 100 cigarettes during their lifetime and they were classified as never/ever/current smokers [25]. In detail, current smokers were defined as having smoked 100 or more cigarettes during their lifetime, participants who met the above criteria before the half year and did not smoke during the past half year were classified as former smokers, and the others were never smokers. Participants reporting their frequency of alcohol consumption at least once per week during the past year were defined as alcohol drinking weekly [26]. We converted the total physical activity level to metabolic equivalent tasks hours/day based on job-related physical activity, transportation physical activity, leisure-time physical activity, and housework [27]. We estimated total energy intake (kcal/week) by summing the number of servings per week of all foods (rice, cooked wheat-based food, vegetables, fruits, meat, etc.), alcohol, tea, oil, and beverages: firstly, we calculated the unit energy of each type of food based on the China Food Composition Tables (2018) and the foods consumption of Guizhou Province; secondly, we further obtained the consumption per week of each type of food based on the data from the food frequency questionnaire; finally, we performed matrix multiplication based on the above two steps. A family history of CVDs was defined as a parent, sibling, or natural child of the participant had a CVDs (coronary heart disease (CHD), stroke, or arrhythmia).

Well-trained examiners took anthropometric and laboratory measurements. We guided all participants to wear light clothing and no shoes and measure twice when measuring body weight and height and the means were used for analysis. We calculated body mass index (BMI) as weight (kg) divided by height (m) squared. Blood pressure was measured three times with a 30-second interval using an electronic sphygmomanometer, with participants in a seated position after five minutes of rest, and they were prohibited to smoke and drink alcohol before measuring their blood pressure. Overnight fasting blood samples after at least eight hours were obtained to assess levels of total cholesterol, triglycerides (TG), high-density lipoprotein-cholesterol, low-density lipoprotein-cholesterol, fasting plasma glucose (FPG), SUA, and Scr. eGFR was calculated using ‘the Xiangya equation’ based on sex, age, and Scr, an estimating equation applied to a multi-ethnic Chinese population [24].

### 2.3. Definition of exposures and outcomes

HUA status was defined by SUA ≥ 7 mg/dL (420 µmol/L) in men and ≥6 mg/dL (360 µmol/L) in women [28]. The primary outcome was composite prevalent CVDs, including CHD, stroke, and arrhythmia, which were identified based on self-reported questionnaire data asking if people had ever been diagnosed by a doctor from second-level and above hospitals with CHD, stroke, or arrhythmia. CVDs could be diagnosed by participants if they had one or more of the conditions. The validity of the self-reported data for CVDs has been verified with an accuracy of approximately 97% and this approach has been used in many population–based studies [29,30,31].

### 2.4. Statistical analyses

Baseline characteristics of participants were reported as frequency and percent, or median and interquartile range. We assessed the significance of differences between CVDs patients and non-CVDs individuals by the Mann-Whitney *U* test for continuous variables and χ^2^ test for categorical variables. The age- and sex-standardized prevalence of CVDs and its subtypes were according to the 2010 China Census data [32].

We used multivariate logistic regression models to explore the potential positive associations of HUA with the risk of CVDs and its subtypes, and multi-adjusted odd ratios (ORs) and 95% confidence intervals (CIs) were reported. We investigated potential sex and age interactions by fitting a multiplicative interaction term into the models between HUA in all three ethnicities, and we found significant interactions for the effect of HUA on CVDs and its subtypes by all of these variables (*P* < 0.05). We conducted stratified analyses by categories of sex (men and women) and age (<median age and ≥ median age) in the Dong, Miao, and Bouyei ethnic groups. All multivariate models were adjusted for the following potential confounders except for the stratified variables: sex, age, residence, tobacco smoking status, alcohol drinking status, total physical activity, total energy intake, family history of CVDs, BMI, and systolic blood pressure, FPG, and TG levels.

All figures were plotted by using STATA version 16 (STATA Corp, College Station, TX) and the other data were analysed with SAS version 9.1 (SAS Inst. Inc., Cary, NC). Statistical significance was set at *P* < 0.05 based on two-sided probability.

## 3. Results

### 3.1. Baseline characteristics

**Figure 1:**
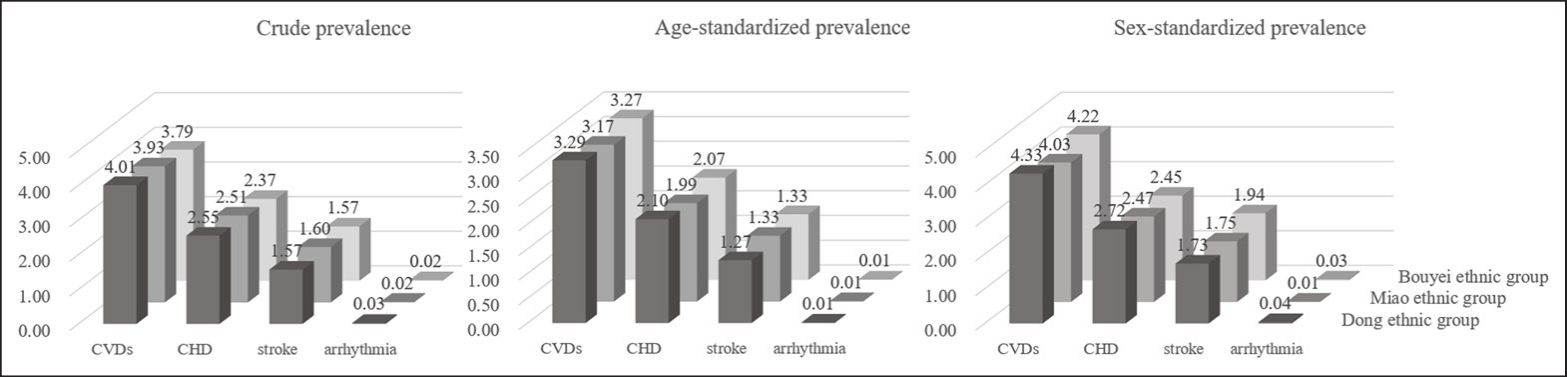
The prevalence of cardiovascular diseases. CHD, coronary heart disease; CVDs, cardiovascular diseases.

In total, 16,618 participants (651 CVDs patients) were included in this study (***Table 1***). The age- and sex-standardized prevalence of CVDs was 3.29% and 4.33%, 3.17% and 4.03%, and 3.27% and 4.22% in the Dong, Miao, and Bouyei ethnic groups, respectively (***Figure 1***). Compared with people without CVDs, CVDs patients were more frequently older, had HUA, and had a lower level of total physical activity and total energy intake but high levels of systolic blood pressure, diastolic blood pressure, SUA, Scr, and eGFR in all three ethnicities; had higher frequency of women and alcohol drinking weekly but a lower high-density lipoprotein-cholesterol level in the Dong ethnic group; and had a higher proportion of rural residence and higher levels of BMI, FPG, TG, and low-density lipoprotein-cholesterol in the Miao and Bouyei ethnic groups (all *P* < 0.05) (***Table 1***).

**Table 1:** Baseline characteristics of 6,228 Dong, 4,986 Miao, and 5,404 Bouyei ethnic groups by cardiovascular diseases status.


VARIABLES	DONG ETHNIC GROUP				MIAO ETHNIC GROUP				BOUYEI ETHNIC GROUP		
		
	NON-CVDS	CVDS	*P*		NON-CVDS	CVDS	*P*		NON-CVDS	CVDS	*P*

**No. of participants**	5,978 (96.00)	250 (4.00)			4,790 (96.07)	196 (3.93)			5,199 (96.21)	205 (3.79)	

**Age (years)**	51.96 (44.99–60.13)	61.11 (54.12–67.00)	<0.001		50.45 (42.51–59.18)	62.08 (53.68–68.63)	<0.001		50.87 (43.89–58.05)	60.16 (53.11–66.77)	<0.001

**Men**	2,052 (34.33)	112 (44.80)	0.001		1,740 (36.33)	79 (40.31)	0.257		1,540 (29.62)	85 (41.46)	<0.001

**Rural**	4,636 (77.84)	188 (75.20)	0.326		3,421 (71.57)	119 (60.71)	0.001		4,554 (87.59)	169 (82.44)	0.029

**Tobacco smoking status**			0.118				0.571				0.736

**Never**	4,761 (79.66)	180 (72.00)			3,748 (78.25)	154 (78.57)			4,301 (82.73)	164 (80.00)	

**Former**	210 (3.51)	28 (11.20)			171 (3.57)	12 (6.12)			161 (3.10)	21 (10.24)	

**Current**	1,006 (16.83)	42 (16.80)			871 (18.18)	30 (15.31)			737 (14.18)	20 (9.76)	

**Alcohol drinking weekly**	188 (3.14)	1 (14.00)	0.032		204 (4.26)	9 (4.59)	0.821		155 (2.98)	8 (3.90)	0.450

**Total physical activity (METs h/d)**	24.96 (14.00–37.16)	17.55 (8.30–30.65)	<0.001		25.18 (13.40–38.80)	12.12 (5.60–27.03)	<0.001		23.95 (13.37–37.10)	14.80 (7.01–28.00)	<0.001

**Total energy intake (kcal/week)**	10.22 (8.20–12.97)	9.24 (7.35–12.08)	<0.001		10.61 (8.16–13.67)	9.71 (7.84–12.23)	0.001		10.65 (8.15–13.81)	9.94 (7.44–12.83)	0.005

**BMI (kg/m^2^)**	23.74 (21.46–26.15)	24.38 (21.81–26.15)	0.224		24.79 (22.51–27.10)	25.50 (22.97–27.80)	0.038		23.84 (21.59–26.20)	24.94 (22.20–27.06)	<0.001

**SBP (mmHg)**	121.60 (110.33–135.67)	129.50 (115.00–144.00)	<0.001		122.30 (111.00–136.67)	134.30 (118.33–147.67)	<0.001		121.30 (110.33–135.67)	135.30 (120.67–151.33)	<0.001

**DBP (mmHg)**	79.00 (72.67–87.00)	81.00 (74.67–90.67)	0.003		80.33 (73.33–88.33)	83.33 (75.67–92.00)	0.001		80.33 (74.00–88.33)	86.00 (78.33–94.67)	<0.001

**FPG (mmol/L)**	5.29 (4.96–5.68)	5.30 (4.94–5.75)	0.643		5.20 (4.90–5.60)	5.45 (5.06–5.92)	<0.001		5.14 (4.86–5.51)	5.28 (5.02–5.73)	<0.001

**TC (mmol/L)**	4.87 (4.29–5.51)	4.84 (4.10–5.52)	0.267		4.96 (4.35–5.60)	5.04 (4.41–5.98)	0.162		4.87 (4.31–5.52)	4.97 (4.41–5.67)	0.081

**TG (mmol/L)**	1.51 (1.08–2.22)	1.58 (1.18–2.39)	0.116		1.43 (1.02–2.12)	1.61 (1.22–2.30)	0.002		1.38 (0.99–2.02)	1.57 (1.16–2.14)	0.002

**HDL-C (mmol/L)**	1.46 (1.22–1.73)	1.37 (1.15–1.62)	<0.001		1.43 (1.02–2.12)	1.43 (1.25–1.60)	0.206		1.49 (1.32–1.69)	1.49 (1.33–1.67)	0.772

**LDL-C (mmol/L)**	2.94 (2.38–3.50)	2.96 (2.26–3.60)	0.824		2.81 (2.30–3.40)	2.90 (2.35–3.61)	0.042		2.45 (2.03–2.93)	2.54 (2.15–3.13)	0.022

**SUA (mg/dL)**	5.36 (4.45–6.52)	5.85 (4.87–7.24)	<0.001		5.39 (4.49–6.45)	5.69 (4.86–7.08)	<0.001		5.02 (4.18–6.08)	5.78 (4.60–6.72)	<0.001

**Scr (µmol/L)**	65.00 (56.00–77.00)	71.00 (59.00–85.00)	<0.001		62.00 (54.00–74.00)	68.00 (59.00–79.50)	<0.001		60.00 (53.00–70.00)	67.00 (57.00–79.00)	<0.001

**eGFR (mL/min/1.73 m^2^)**	81.00 (75.00–87.00)	76.00 (70.00–82.00)	<0.001		84.00 (77.00–90.00)	76.00 (71.00–82.00)	<0.001		84.00 (78.00–90.00)	78.00 (72.00–84.00)	<0.001

**HUA**	1,545 (25.84)	92 (36.80)	<0.001		1,189 (24.82)	66 (33.67)	0.005		969 (18.64)	66 (32.20)	<0.001


Abbreviations: BMI, body mass index; CVDs, cardiovascular diseases; DBP, diastolic blood pressure; eGFR, estimated glomerular filtration rate; FPG, fasting plasma glucose; HDL-C, high-density lipoprotein cholesterol; HUA, hyperuricemia; LDL-C, low-density lipoprotein cholesterol; METs h/d, metabolic equivalent tasks hours/day; SBP, systolic blood pressure; Scr, serum creatinine; SUA, serum uric acid; TC, total cholesterol; TG, triglycerides. Data are number (percentage) or median (interquartile range). *P* comparing non-CVDs and CVDs.

### 3.2. The risk CVDs, CHD, and stroke stratified by sex

**Figure 2:**
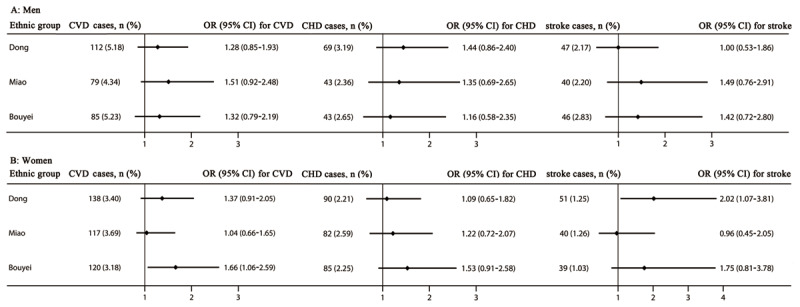
The risk of cardiovascular diseases, coronary heart disease, and stroke stratified by sex. Adjusted for age, residence, tobacco smoking status, alcohol drinking status, total physical activity, total energy intake, family history of cardiovascular diseases, body mass index, and systolic blood pressure, fasting plasma glucose, and triglycerides levels. CHD, coronary heart disease; CI, confidence interval; CVDs, cardiovascular diseases; OR, odd ratio.

In the sex-specific analysis, multivariate logistic regression analysis after adjustment for potential risk factors revealed that HUA was positively associated with the risk of CVDs in women in the Bouyei ethnic group, and adjusted OR (95% CI) was 1.66 (1.06–2.59). Additionally, a 2.02-fold increased risk for stroke in women in the Dong ethnic group was found. However, we did not observe a significant association in men in the three ethnic groups (***Figure 2***).

### 3.3. The risk of CVDs, CHD, and stroke stratified by the median of age

**Figure 3:**
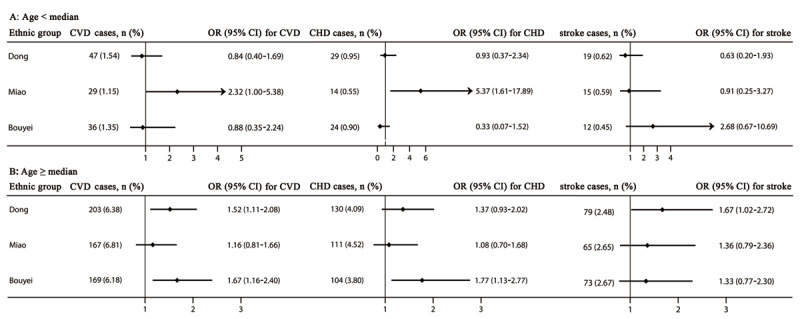
The risk of cardiovascular diseases, coronary heart disease, and stroke stratified by the median of age. Adjusted for sex, residence, tobacco smoking status, alcohol drinking status, total physical activity, total energy intake, family history of cardiovascular diseases, body mass index, and systolic blood pressure, fasting plasma glucose, and triglycerides levels. CHD, coronary heart disease; CI, confidence interval; CVDs, cardiovascular diseases; OR, odd ratio. The median of age were 52, 51, and 51 years in the Dong, Miao, and Bouyei ethnic groups, respectively.

After adjusting for potential confounders, among people aged less than the median of age, a 2.32-fold (OR 2.32, 95% CI 1.00–5.38) and 5.37-fold (5.37, 1.61–17.89) increase in the risk of CVDs and CHD related to HUA was observed in the Miao ethnic group (***Figure 3***). Among people aged greater than or equal to the median of age, we found a 52% (1.52, 1.11–2.08) and 67% (1.67, 1.02–2.72) increased risk for CVDs and stroke in the Dong ethnic group; moreover, a 67% (1.67, 1.16–2.40) and 77% (1.77, 1.13–2.77) increased risk for CVDs and CHD in the Bouyei ethnic group was detected (***Figure 3***).

### 3.4. The risk CVDs, CHD, and stroke stratified by sex and the median of age

**Figure 4:**
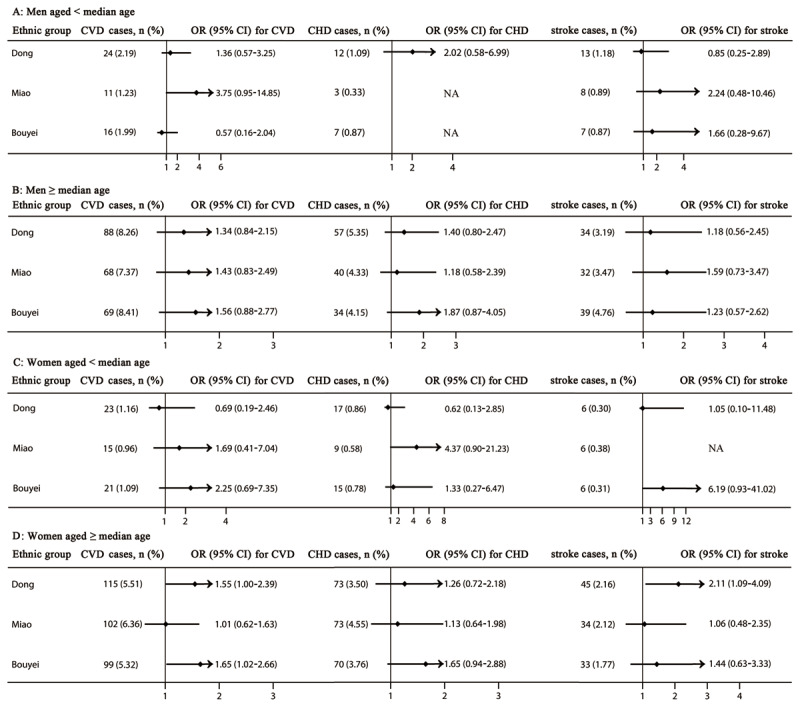
The risk of cardiovascular diseases, coronary heart disease, and stroke stratified by sex and the median of age. Adjusted for residence, tobacco smoking status, alcohol drinking status, total physical activity, total energy intake, family history of cardiovascular diseases, body mass index, and systolic blood pressure, fasting plasma glucose, and triglycerides levels. CHD, coronary heart disease; CI, confidence interval; CVDs, cardiovascular diseases; NA, not applicable; OR, odd ratio. The median of age were 54, 52, and 52 years in men, 51, 50, and 51 years in women, in the Dong, Miao, and Bouyei ethnic groups, respectively.

The risk of CVDs was associated with HUA among women aged greater than or equal to the median of age in the Dong and Bouyei ethnic groups but not the Miao ethnic group considering possible confounding factors, multi-adjusted OR (95% CI) 1.55 (1.00–2.39) and 1.65 (1.02–2.66), respectively. Additionally, the risk of stroke increased with HUA, OR (95% CI) 2.11 (1.09–4.09) (***Figure 4***).

## 4. Discussion

Among 16,618 participants (6,228 Dong, 4,986 Miao, and 5,404 Bouyei), which included 651 CVDs patients (250 Dong, 196 Miao, and 205 Bouyei) with a normal level of eGFR in the ethnic Chinese groups aged 30–79 years, we found ethnic differences in the relationship between HUA and the risk of CVDs. HUA was positively associated with 55% and 65% risk increases in CVDs among women ≥ median age in the Dong and Bouyei ethnic groups but not the Miao ethnic group.

A detailed risk assessment of CVDs associated with HUA (definite definition) in previous studies is not well documented [7,9,10,11,17,18]. Furthermore, most of them focused primarily on total populations [8,11,12,13,14,15] and whether sex has an effect on the relationship remains unclear [7,9,10,16,17,18]. In the current study, a positive relationship between HUA and the risk of CVDs existed in women in the Dong and Bouyei ethnic groups but not among men or the Miao ethnic group. Sex differences in the relationship could be related to intrinsic biologic differences or differences in risk factor management. Sex hormones, visceral adiposity, and muscle mass have sex-specific changes, which have been reported to regulate SUA metabolism. Additionally, there may be differences in gene expression patterns among the three ethnic groups.

In addition, it is still unclear whether the changes of SUA with age will affect the relationship [9,10,15,16,17]. In our study population, those ≥ median age with HUA had an increased risk of CVDs in the Dong and Bouyei ethnic groups; however, a higher risk of CVDs was found in people aged < median age in the Miao ethnic group. Using SUA-lowering drugs in HUA patients at earlier stages of the disease could contribute to the risk reduction in CVDs. The Miao ethnic group may put more emphasis on the control of SUA level in young or middle age than the Dong and Bouyei ethnic groups and thus the HUA associated with CVDs risk could be reduced. Also, the use of CVDs medications could lead to an increase in the level of SUA and further affect the HUA–CVDs relationship. Additionally, relatively unhealthy and unreasonable diet habits, including higher consumption of high purine foods by young and middle-aged adults than among elderly people could affect the level of SUA. As well, the low metabolic capacity of elderly people may contribute to increases in the SUA level.

As people age, the HUA-related risk of cardiometabolic diseases might be increased. Those with HUA could have extensive vascular damage and need to maintain adequate organ perfusion. HUA may result in reduced nitric oxide availability, increased oxidative stress, endothelial dysfunction, inflammation promotion, or insulin resistance [33,34]. The proliferation of vascular smooth cells, vascular inflammation, or atherosclerosis may be induced or accelerated by lipid peroxidation and platelet adhesion related to HUA [35,36]. Atherosclerosis may be caused by renal impairment related to HUA and could induce homocysteinemia [37]. The above-mentioned possible mechanisms are more serious in elderly people, which could be why older participants are at higher risk of CVDs related to HUA compared with middle-aged people. Notably, possible ethnic differences in their living and diet habits may be influential in the HUA–CVDs relationship.

Stratified analyses combined by sex and age have rarely been performed [9]. Only one observational study that included 8,285 adults from northeastern China was performed to assess the association of HUA with the risk of CHD, and it found a positive relationship in women aged ≥ 80 years [9]. The present study suggested that HUA was related to the risk of CVDs in women aged ≥ median age in the Dong and Bouyei ethnic groups. Considering the above-mentioned potential explanations, a combined effect of intrinsic biologic differences, consumption of high purine foods, low metabolic capacity, and SUA-lowering drugs on the HUA–CVDs relationship could be observed in elderly women. Further investigation of the underlying mechanisms is necessary.

The observed relationship between HUA and the risk of CVDs was considerable. If causal, a moderately weak or strong association may be relevant to ‘Health China 2030.’ For example, if good primary prevention and control of HUA were achieved, it could be expected that the CVDs risk might consequently be reduced by at least 55% based on the present findings. CVDs are the leading cause of death, especially women [38], and a small percentage reduction in HUA-related CVDs risk will have a significant public health impact.

## 5. Strengths and limitations

The primary strength of this study was the multi-ethnic Chinese population. Moreover, we performed stratified analysis by sex and age and considered the effect of kidney function on the association. Additionally, we calculated the eGFR level based on an estimating equation applied to a multi-ethnic Chinese population.

Some limitations warrant consideration when interpreting the findings of this study. First, this study was conducted on participants from the three ethnic groups in one area. We could not evaluate the HUA–CVDs relationship in other ethnicities or the included ethnicities in the present study from other areas. Second, it is difficult to assess the causal association of HUA and the risk of CVDs in consideration of the cross-sectional design. Third, the levels of SUA and Scr were only measured once, and intraindividual variability was not taken into account. Fourth, the average age of the participants was older, and larger samples are need. Fifth, detailed categories of age, the subtypes of CHD and stroke, and arrhythmia were not analysed because of the limited number of cases. Finally, there is the possibility of residual confounding factors in this observational study because of some unmeasured or unknown covariates.

## 6. Conclusions

In conclusion, elderly women in the Dong and Bouyei ethnic groups in China had an increased risk of CVDs associated with HUA. Given the suggestive findings observed in the present study, future multi-ethnic, sex-specific, and age-specific studies are warranted to perform detailed and causal assessments to determine the potential mechanisms. Until the evidence is externally validated, however, most patients with HUA, especially elderly women, will still require control of their SUA level to reduce their potential risk of CVDs.

## Data Accessibility Statement

The data that support the findings of this study are available from the corresponding author upon reasonable request.

## References

[1] GBD 2019 Diseases and Injuries Collaborators. Global burden of 369 diseases and injuries in 204 countries and territories, 1990–2019: A systematic analysis for the Global Burden of Disease Study 2019. LANCET. 2020; 396: 1204–22. DOI: 10.1016/S0140-6736(20)30925-9 33069326 PMC7567026

[2] Zhao D. Epidemiological Features of Cardiovascular Disease in Asia. JACC: Asia. 2021; 1: 1–13. DOI: 10.1016/j.jacasi.2021.04.007 PMC9627928 36338365

[3] The Writing Committee of the Report on Cardiovascular Health and Diseases in China. Interpretation of Report on Cardiovascular Health and Diseases in China 2020. CHIN J CARDIOVASC MED. 2021; 26: 209–218. DOI: 10.3969/j.issn.1007-5410.2021.03.001

[4] GBD 2019 Stroke Collaborators. Global, regional, and national burden of stroke and its risk factors, 1990–2019: A systematic analysis for the Global Burden of Disease Study 2019. LANCET NEUROL. 2021; 20: 795–820. DOI: 10.1016/S1474-4422(21)00252-0 34487721 PMC8443449

[5] Dagenais GR, Leong DP, Rangarajan S, et al. Variations in common diseases, hospital admissions, and deaths in middle-aged adults in 21 countries from five continents (PURE): A prospective cohort study. LANCET. 2020; 395: 785–94. DOI: 10.1016/S0140-6736(19)32007-0 31492501

[6] Su JT, Zhang YM, Wang P, Du J, Wei ZH. Comparative analysis of comprehensive health status among 31 provinces in China and 134 countries (regions) in 2015. ZHŌNGHUÁ YÙFÁNG-YĪXUÉ ZÁZHÌ. 2020; 54: 165–8. DOI: 10.3760/cma.j.issn.0253-9624.2020.02.010. 32074704

[7] Chilunga FP, Henneman P, Requena Méndez A, et al. Hyperuricaemia and its association with 10-year risk of cardiovascular disease among migrant and non-migrant African populations: the RODAM study. TROP MED INT HEALTH. 2020; 25: 496–505. DOI: 10.1111/tmi.13362 31825117

[8] Chaudhary NS, Bridges SL, Saag KG, et al. Severity of Hypertension Mediates the Association of Hyperuricemia With Stroke in the REGARDS Case Cohort Study. HYPERTENSION. 2020; 75: 246–56. DOI: 10.1161/HYPERTENSIONAHA.119.13580 31786980 PMC7122733

[9] Sun Y, Zhang H, Tian W, et al. Association between serum uric acid levels and coronary artery disease in different age and gender: A cross-sectional study. AGING CLIN EXP RES. 2019; 31: 1783–90. DOI: 10.1007/s40520-019-01137-2 30694512

[10] Tu W, Wu J, Jian G, et al. Asymptomatic hyperuricemia and incident stroke in elderly Chinese patients without comorbidities. EUR J CLIN NUTR. 2019; 73: 1392–402. DOI: 10.1038/s41430-019-0405-1 30787471

[11] Lai X, Yang L, Légaré S, et al. Dose-response relationship between serum uric acid levels and risk of incident coronary heart disease in the Dongfeng-Tongji Cohort. INT J CARDIOL. 2016; 224: 299–304. DOI: 10.1016/j.ijcard.2016.09.035 27665401

[12] Navaneethan SD, Beddhu S. Associations of serum uric acid with cardiovascular events and mortality in moderate chronic kidney disease. NEPHROL DIAL TRANSPL. 2008; 24: 1260–6. DOI: 10.1093/ndt/gfn621 PMC2721426 19033255

[13] Chuang S, Chen J, Yeh W, Wu C, Pan W. Hyperuricemia and increased risk of ischemic heart disease in a large Chinese cohort. INT J CARDIOL. 2012; 154: 316–21. DOI: 10.1016/j.ijcard.2011.06.055 21862159

[14] Chien K, Hsu H, Sung F, Su T, Chen M, Lee Y. Hyperuricemia as a risk factor on cardiovascular events in Taiwan: The Chin-Shan Community Cardiovascular Cohort Study. ATHEROSCLEROSIS. 2005; 183: 147–55. DOI: 10.1016/j.atherosclerosis.2005.01.018 16154134

[15] Wang H, Jacobs DR, Gaffo AL, Gross MD, Goff DC, Carr JJ. Serum Urate and Incident Cardiovascular Disease: The Coronary Artery Risk Development in Young Adults (CARDIA) Study. PLOS ONE. 2015; 10: e138067. DOI: 10.1371/journal.pone.0138067 PMC4575092 26381512

[16] Wu J, Lei G, Wang X, et al. Asymptomatic hyperuricemia and coronary artery disease in elderly patients without comorbidities. ONCOTARGET. 2017; 8: 80688–99. DOI: 10.18632/oncotarget.21079 29113336 PMC5655231

[17] Musacchio E, Perissinotto E, Sartori L, et al. Hyperuricemia, Cardiovascular Profile, and Comorbidity in Older Men and Women: The Pro.V.A. Study. REJUV RES. 2017; 20: 42–9. DOI: 10.1089/rej.2016.1834 27241310

[18] Yang Y, Tian J, Zeng C, et al. Relationship between hyperuricemia and risk of coronary heart disease in a middle-aged and elderly Chinese population. J INT MED RES. 2017; 45: 254–60. DOI: 10.1177/0300060516673923 28222629 PMC5536609

[19] Zhao X, Hong F, Yin J, et al. Cohort profile: the China Multi-Ethnic cohort (CMEC) study. INT J EPIDEMIOL. 2020; 50: 721–721l. DOI: 10.1093/ije/dyaa185 PMC8271196 33232485

[20] Xu H, Guo B, Qian W, et al. Dietary Pattern and Long-Term Effects of Particulate Matter on Blood Pressure: A Large Cross-Sectional Study in Chinese Adults. HYPERTENSION. 2021; 78: 184–94. DOI: 10.1161/HYPERTENSIONAHA.121.17205 33993725

[21] Zhang N, Xiao X, Xu J, et al. Dietary Approaches to Stop Hypertension (DASH) diet, Mediterranean diet and blood lipid profiles in less-developed ethnic minority regions. BRIT J NUTR. 2021; 1–25. DOI: 10.1017/S0007114521004013 34605387

[22] Wang Y, Zeng Y, Zhang X, et al. Daytime Napping Duration Is Positively Associated With Risk of Hyperuricemia in a Chinese Population. J CLIN ENDOCR METAB. 2021; 106: e2096–105. DOI: 10.1210/clinem/dgab043 33507274

[23] Wang L, Chen G, Pan Y, et al. Association of long-term exposure to ambient air pollutants with blood lipids in Chinese adults: The China Multi-Ethnic Cohort study. ENVIRON RES. 2021; 197: 111174. DOI: 10.1016/j.envres.2021.111174 33894235

[24] Li D, Yin W, Yi Y, et al. Development and validation of a more accurate estimating equation for glomerular filtration rate in a Chinese population. KIDNEY INT. 2019; 95: 636–46. DOI: 10.1016/j.kint.2018.10.019 30709663

[25] Liu X, Bragg F, Yang L, et al. Smoking and smoking cessation in relation to risk of diabetes in Chinese men and women: A 9-year prospective study of 0.5 million people. LANCET PUBLIC HEALTH. 2018; 3: e167–76. DOI: 10.1016/S2468-2667(18)30026-4 29548855 PMC5887081

[26] Millwood IY, Li L, Smith M, et al. Alcohol consumption in 0.5 million people from 10 diverse regions of China: prevalence, patterns and socio-demographic and health-related correlates. INT J EPIDEMIOL. 2017; 46: 2103. DOI: 10.1093/ije/dyx210 29025163 PMC5837380

[27] Du H, Bennett D, Li L, et al. Physical activity and sedentary leisure time and their associations with BMI, waist circumference, and percentage body fat in 0.5 million adults: The China Kadoorie Biobank study. AM J CLIN NUTR. 2013; 97: 487–96. DOI: 10.3945/ajcn.112.046854 23364014 PMC4345799

[28] Chinese Society of Endocrinology. Chinese Guideline of Hyperuricemia and Gout. CHIN J ENDOCRINOL METAB. 2013; 29: 913–20. DOI: 10.3760/cma.j.issn.1673-4157.2013.06.005

[29] Yang B, Guo Y, Morawska L, et al. Ambient PM1 air pollution and cardiovascular disease prevalence: Insights from the 33 Communities Chinese Health Study. ENVIRON INT. 2019; 123: 310–7. DOI: 10.1016/j.envint.2018.12.012 30557810

[30] Davies NM, Dickson M, Davey SG, van den Berg GJ, Windmeijer F. The Causal Effects of Education on Health Outcomes in the UK Biobank. NAT HUM BEHAV. 2018; 2: 117–25. DOI: 10.1038/s41562-017-0279-y 30406209 PMC6217998

[31] Qin XD, Qian Z, Vaughn MG, et al. Gender-specific differences of interaction between obesity and air pollution on stroke and cardiovascular diseases in Chinese adults from a high pollution range area: A large population based cross sectional study. SCI TOTAL ENVIRON. 2015; 529: 243–8. DOI: 10.1016/j.scitotenv.2015.05.041 26022408

[32] National Bureau of Statistics of China. 2010 Population Census. Retrieved from: http://www.stats.gov.cn/tjsj/pcsj/rkpc/6rp/indexch.htm. (Accessed 1 June 2021).

[33] Sharaf El Din UAA, Salem MM, Abdulazim DO. Uric acid in the pathogenesis of metabolic, renal, and cardiovascular diseases: A review. J ADV RES. 2017; 8: 537–48. DOI: 10.1097/HJH.0b013e3282f240bf 28748119 PMC5512153

[34] Khosla UM, Zharikov S, Finch JL, et al. Hyperuricemia induces endothelial dysfunction. KIDNEY INT. 2005; 67: 1739–42. DOI: 10.1111/j.1523-1755.2005.00273.x 15840020

[35] Corry DB, Eslami P, Yamamoto K, Nyby MD, Makino H, Tuck ML. Uric acid stimulates vascular smooth muscle cell proliferation and oxidative stress via the vascular renin-angiotensin system. J HYPERTENS. 2008; 26: 269–75. DOI: 10.1097/HJH.0b013e3282f240bf 18192841

[36] Ruggiero C, Cherubini A, Ble A, et al. Uric acid and inflammatory markers. EUR HEART J. 2006; 27: 1174–81. DOI: 10.1093/eurheartj/ehi879 16611671 PMC2668163

[37] Park JH, Song JS, Choi ST. Increased Carotid Intima-Media Thickness (IMT) in Hyperuricemic Individuals May Be Explained by Hyperhomocysteinemia Associated with Renal Dysfunction: A Cross-Sectional Study. J KOREAN MED SCI. 2019; 34: e237. DOI: 10.3346/jkms.2019.34.e237 31559709 PMC6763401

[38] Vogel B, Acevedo M, Appelman Y, et al. The Lancet women and cardiovascular disease Commission: reducing the global burden by 2030. LANCET. 2021; 397: 2385–438. DOI: 10.1016/S0140-6736(21)00684-X 34010613

